# Expression of the *foraging* gene in adult *Drosophila melanogaster*

**DOI:** 10.1080/01677063.2021.1941946

**Published:** 2021-08-12

**Authors:** Aaron M. Allen, Marla B. Sokolowski

**Affiliations:** a Department of Cell and Systems Biology, University of Toronto, Toronto, Canada; b Centre for Neural Circuits and Behaviour, University of Oxford, Oxford, UK; c Department of Ecology and Evolutionary Biology, University of Toronto, Toronto, Canada; d Child and Brain Development Program, Canadian Institute for Advanced Research (CIFAR), Toronto, Canada

**Keywords:** Drosophila melanogaster, foraging gene, expression, promoter analysis, cis-regulatory element, pleiotropy

## Abstract

The *foraging* gene in *Drosophila melanogaster*, which encodes a cGMP-dependent protein kinase, is a highly conserved, complex gene with multiple pleiotropic behavioral and physiological functions in both the larval and adult fly. Adult *foraging* expression is less well characterized than in the larva. We characterized *foraging* expression in the brain, gastric system, and reproductive systems using a *T2A-Gal4* gene-trap allele. In the brain, *foraging* expression appears to be restricted to multiple sub-types of glia. This glial-specific cellular localization of *foraging* was supported by single-cell transcriptomic atlases of the adult brain. *foraging* is extensively expressed in most cell types in the gastric and reproductive systems. We then mapped multiple *cis*-regulatory elements responsible for parts of the observed expression patterns by a nested cloned promoter-*Gal4* analysis. The mapped *cis*-regulatory elements were consistently modular when comparing the larval and adult expression patterns. These new data using the *T2A-Gal4* gene-trap and cloned *foraging* promoter fusion *GAL4*’s are discussed with respect to previous work using an anti-FOR antibody, which we show here to be non-specific. Future studies of *foraging*’s function will consider roles for glial subtypes and peripheral tissues (gastric and reproductive systems) in *foraging*’s pleiotropic behavioral and physiological effects.

## Introduction

Genes that influence behavior are often pleiotropic, expressed throughout development and in many tissues (Hall, 1994). Investigating the temporal and spatial expression of pleiotropic genes can aid in the interpretation of their many functions. The *Drosophila melanogaster foraging (for)* gene, which codes for a cGMP-dependent protein kinase (PKG), has long been a model system for studies of gene–environment interaction and pleiotropy in the field of behavior genetics (Allen, Anreiter, Neville, & Sokolowski, 2017; de Belle, Hilliker, & Sokolowski, 1989; Osborne *et al.*, 1997; Sokolowski, 1980; reviewed in Anreiter & Sokolowski, 2019). *foraging* is a complex gene with a modular structure that includes four promoters (*for^pr1-4^*), which produce approximately 20 RNA transcripts that code for nine distinct protein isoforms (Allen *et al.*, 2017). The different isoforms of *foraging* have varied expression levels depending on the developmental stage and tissue being assayed (Allen *et al.*, 2017; Anreiter, Kramer, & Sokolowski, 2017; Brown *et al.*, 2014; Dason *et al.*, 2020; Leader, Krause, Pandit, Davies, & Dow, 2018), but little is known about the cell-specific expression of *foraging*.

Some indication of the tissue-specific requirement for a suite of feeding-related and physiological phenotypes has been described in the larva (Allen, Anreiter, Vesterberg, Douglas, & Sokolowski, 2018; Dason, Allen, Vasquez, & Sokolowski, 2019, Dason & Sokolowski, 2021). We previously generated a full genetic deletion of the *foraging* gene, *for^0^*, and found that these *foraging* null larvae had reduced locomotion on food, reduced food intake, and increased triglyceride content (Allen *et al.*, 2017). We were then able to rescue these attributes using different cloned *foraging* regulatory regions, which drove expression in discrete and restricted patterns in third instar larvae (Allen *et al.*, 2018). Specifically, the decrease in larval locomotion on food of the *for^0^* mutant was rescued by *for^pr1^-Gal4* when driving *UAS-for^cDNA^*. *for^pr1^-Gal4* expressed in neurons of the CNS and enteroendocrine cells (EE cells) of the gut. Triglyceride levels were rescued by *for^pr3^-Gal4*, which expressed in many cell types such as perineurial glia, visceral muscle, trachea, and fat cells (among others). Finally, food intake was rescued by *for^pr4^-Gal4*, which expressed in the developing optic lobes, hindgut, and spiracles (Allen *et al.*, 2018). Similar tissue-specific requirements of *foraging* were found at the larval neuromuscular junction where the *for^0^* null mutant phenotype of nerve terminal overgrowth was rescued by glial expression, and increased neurotransmission was rescued by neuronal expression (Dason *et al.*, 2019). Hence, these discrete and restricted expression patterns were successfully used to dissect some of the pleiotropic behavioral and physiological functions of *foraging* during the larval stage.

In addition to its larval phenotypes, the *foraging* gene has been implicated in many adult behavioral phenotypes. These include post-feeding locomotion (Pereira & Sokolowski, 1993), sucrose responsiveness (Belay *et al.*, 2007; Scheiner, Sokolowski, & Erber, 2004), learning and memory (Kaun *et al.*, 2007; Kohn *et al.*, 2013; Kuntz, Poeck, Sokolowski, & Strauss, 2012; Mery, Belay, So, Sokolowski, & Kawecki, 2007; Reaume, Sokolowski, & Mery, 2011; Wang *et al.*, 2008), habituation (Eddison, Belay, Sokolowski, & Heberlein, 2012; Engel, Xie, Sokolowski, & Wu, 2000; Scheiner *et al.*, 2004), social behavior (Donlea *et al.*, 2012; Foucaud *et al.*, 2013; Alwash *et al.*, 2021), sleep (Donlea *et al.*, 2012), starvation resistance (Anreiter *et al.*, 2017; Donlea *et al.*, 2012), aggression (Wang & Sokolowski, 2017), and stress tolerance (Dawson-Scully, Armstrong, Kent, Robertson, & Sokolowski, 2007; Dawson-Scully *et al.*, 2010). Many of these phenotypes show parallels between the larva and the adult developmental stages. For instance, *foraging* affects food search behavior in both larvae and adults (Anreiter *et al.*, 2017; Hughson *et al.*, 2018; Kent, Daskalchuk, Cook, Sokolowski, & Greenspan, 2009; Pereira & Sokolowski, 1993; Sokolowski & Riedl, 1999). However, the behavioral patterns seen for food intake are reversed when comparing larvae and adults. Larvae carrying the *for^0^* null allele show decreased food intake relative to controls, and this can be rescued by a full transgenic complement of the locus as well with a tissue-specific expression of a *foraging* cDNA (Allen *et al.*, 2017, 2018). In contrast, food deprived adults with *for^pr4^ foraging* transcripts ubiquitously knocked-down with RNAi consume more sucrose drops than controls (Anreiter *et al.*, 2017). This suggests the possibility of differential regulation of the *foraging* gene for specific phenotypes at the larval and adult developmental stages. In the larva, *foraging*’s pleiotropy is in part due to *foraging*’s four promoters driving distinct expression patterns of subsets of transcripts, each associated with different phenotypes. We wondered whether *foraging* exhibited promoter-specific expression patterns in the adult and if they paralleled those observed in larvae.

Here we characterize the adult expression of *foraging* in the brain, gastric system, and reproductive systems, using: a) a transcriptional and translational trap *T2A-Gal4* allele (Lee *et al.*, 2018), b) published data from single-cell transcriptomic analyses (Davie *et al.*, 2018; Hung *et al.*, 2020), c) *foraging* promoter *Gal4* fusions, and d) by revisiting a previously published anti-FOR antibody. We further refine and map regions containing *cis*-regulator elements using a series of nested promoter *Gal4* fusions and compare the modular patterns of expression of the larval and adult fly.

## Materials and methods

### Fly strains and rearing

Flies were reared in 40 ml vials with 10 ml of food with a 12 L:12D photocycle at 25 ± 1 °C. The food recipe has previously been described (Allen *et al.*, 2017). The following fly strains were used in this study: *for^0^/CyO, {Act-GFP}* (Allen *et al.*, 2018), *{for^pr1^-Gal4}attP2* (Allen *et al.*, 2018), *{for^pr2^-Gal4}attP2* (Allen *et al.*, 2018), *{for^pr3^-Gal4}attP2* (Allen *et al.*, 2018), *{for^pr4^-Gal4}attP2* (Allen *et al.*, 2018), y^1^,w*; TI{GFP[3xP3.cLa]=CRIMIC.TG4.2}for^CR00867-TG4.2^ (Bloomington Drosophila Stock Center # 79329; Lee *et al.*, 2018), w*; P{UAS-mCD8::GFP}/CyO (denoted as mCD8::GFP), *w^1118^; P{UAS-Stinger}2* (Bloomington Drosophila Stock Center # 84277, denoted as nls::GFP; Barolo, Carver, & Posakony, 2000), w*; UAS-myr-GFP-V5-P2A-H2B-mCherry-HA/TM3, Ser (denoted as *UAS-Watermelon*; Chang, Keegan, Prazak, & Dubnau, 2019).

### Construction of nested Gal4s

Construction of the *for^pr^-Gal4*s was previously described (Allen *et al.*, 2018). A similar strategy was used to generate the nested *for^prΔ^-Gal4*s constructs. The *Not*I fragment, containing the *Gal4* sequence, of pMARTINI-*Gal4* (Billeter & Goodwin, 2004) was cloned into the *Not*I digested pStinger-*attB* vector, replacing the GFP. This insulated *Gal4* vector with *attB* was then digested with *Kpn*I and end filled with Klenow (cat # M0210S, New England Biolabs). Nested regions of *foraging* were amplified by PCR from the larger *for^pr^-Gal4* vectors and cloned into the end filled insulated *Gal4* vector. All *for^prΔ^-Gal4*s constructs were injected into the *P{CaryP}attP2* landing site (Groth, Fish, Nusse, & Calos, 2004) by Genetic Services Inc. (Cambridge, MA, USA). Successful integration was confirmed with PCR.

The primers used for the nested *for^prΔ^-Gal4*s are as follows:

- *for^pr1Δ1^*-F: 5′-TCGCAAAAACCAACCCTTAC-3′,
- *for^pr1Δ2^*-F: 5′-CGACGAACATTATTTGGCTCT-3′,
- *for^pr1Δ3^*-F: 5′-CCTTTCTCCCAGCTGCTATCT-3′,
- *for^pr1Δ4^*-F: 5′-CAAAGTTAATCCTGCATTGGC-3′,
- *for^pr1^*-R: 5′-ACAAGTCGATGAAAAACCGCC-3′,
- *for^pr2Δ1^*-F: 5′-CTAAACGTTTTCCGCAGCA-3′,
- *for^pr2Δ2^*-F: 5′-ACAAACGAATGGAACGGAAC-3′,
- *for^pr2^*-R: 5′-CCAAAACCAAGTGTAACACAC-3′,
- *for^pr3Δ1^*-F: 5′-ATACCCTCCATCCAAAGCG-3′,
- *for^pr3Δ2^*-F: 5′-TCCAAACGGATCTTTGTCTTTT-3′,
- *for^pr3Δ3^*-F: 5′-CAGGGGAAATGATAACCGAA-3′,
- *for^pr3Δ4^*-F: 5′-GCACATAGAACCCGTAGAGGA-3′,
- *for^pr3^*-R: 5′-GGGATCCTGGTTCAATTGCTG-3′,
- *for^pr4Δ1^*-F: 5′-CCCTACTCATAAAACTGCCCC-3′,
- *for^pr4Δ2^*-F: 5′-AGTTCGCCGGTTTGGTACT-3′,
- *for^pr4Δ3^*-F: 5′-TTTTCGCTCTCCCAGACACAC-3′,
- *for^pr4^*-R: 5′-CGAATTGAAAATCACGATACG-3′.

### Recombineering BAC{for^IRES-Gal4^} allele

We generated a transgenic copy of the entire *foraging* locus, replacing the common coding region with a *Gal4* coding sequence, using recombineering. We used the BAC containing the entire 35 kb *foraging* locus (previously described in Allen *et al.*, 2017) and replaced the common coding region of *foraging* with a premature stop codon, followed by an *Internal-Ribosomal-Entry-Site* (*IRES*) and codon optimized *Gal4* coding sequence. The *Gal4* coding sequence replaced the *foraging* common coding sequence, but still utilized the endogenous *foraging* 3’UTR.

To generate this *BAC{for^IRES-Gal4^}* allele we needed to clone a donor construct with *Gal4* sequence flanked with homology arms to *foraging*. Restriction sites added to primers are in parentheses and their sequences are italicized and separated by a hyphen. A *D. melanogaster* codon optimized *Gal4* sequence was PCR amplified, with primers dmGal4-F (*Asc*I) 5′-*GGCGCGCC*-ATGAAGCTGCTGAGTAGTATTG-3′ and dmGal4-R (*Sbf*I) 5′-*CCTGCAGG*-CTACTCCTTCTTTGGGTTCGG-3′, from the pBPGAL4.1Uw vector (Pfeiffer *et al.*, 2010; addgene # 26226) and cloned into the pSC-A-amp/kan vector from the StrataClone PCR Cloning Kit (Agilent, cat # 240205). The *Spe*I–*Not*I fragment containing an *FRT-kan-FR*T cassette from the pIGCN21 vector (Lee *et al.*, 2001) was cloned into the *Spe*I and *Not*I sites of the pSC-dmGAl4 vector. An *Internal-Ribosomal-Entry-Site* (*IRES*) was PCR amplified from the *Ubx* locus (as in Halfon *et al.*, 2002) with the primers Stop-IRES-F (*Hind*III) 5′-*AAGCTT*-CTAGACTAG-TCTAGCAGCAAAGTGCAATTGGCTAAAAACC-3′ and Stop-IRES-R (*Asc*I) 5′-*GGCGCGCC*-GATTCTTACCGCCAGCAGCGC-3′ and cloned into the *Hind*III and *Asc*I sties of the pSC-dmGAl4-FRT-kan-FRT vector. An all 6 frame stop codon cassette added to the forward primer is underlined and separated by a hyphen. A left homology arm corresponding to *foraging* specific sequence was PCR amplified with L-comGal4-F (*Kpn*I) 5′-*GGTACC*-GCTCCGCCACCCAGAGAACC-3′ and L-comGal4-R (*Hind*III) 5′-*AAGCTT*-CCTCGCGGGAAACCTCCACG-3′ and cloned into the *Kpn*I and *Hind*III sites of the pSC-IRES-dmGAl4-FRT-kan-FRT vector. A right homology arm corresponding to *foraging* specific sequence was PCR amplified with R-comGal4-F (*Bgl*II) 5′-*AGATCT*-GGAGAATCAGAACCCGTTTC-3′ and R-comGal4-R (*Not*I) 5′-*GCGGCCGC*-GCATACAAATCGGGTTGCCTT-3′ and cloned in the *Bgl*II and *Not*I sites of the pSC-LHA-IRES-dmGAl4-FRT-kan-FRT vector.

The *Kpn*I–*Not*I fragment from the pSC-LHA-IRES-dmGAl4-FRT-kan-FRT-RHA vector was transformed into EL250 *E. coli* strain (Lee *et al.*, 2001) which already contained a bacterial artificial chromosome (BAC) containing the 35 kb *foraging* locus (previously described in Allen *et al.*, 2017). Recombineered BACs were selected by kanamycin resistance. The *FRT-kan-FRT* was then removed by arabinose induction. Proper integration *Gal4* sequence and replacement of the *foraging* common coding region was verified with PCR, restriction digest, and Sanger sequencing. φC31 integration was used to integrate the BAC into the *VK00013* landing site on the third chromosome (Venken, He, Hoskins, & Bellen, 2006). Transgenesis was performed by BestGene Inc. Primer design, in-slico cloning, and analysis of Sanger sequencing reactions were all performed in the Geneious 8 software package (Kearse et al., 2012).

### Immunohistochemistry

Adult and larval samples were dissected in 1 × PBS and then fixed in 4% paraformaldehyde in 1× PBS for 40 min. The tissues were then rinsed twice and then washed 4× for 45 min each in 0.3% Triton X in 1× PBS (PBT). The tissues were blocked in 10% normal goat serum (NGS, Jackson ImmunoResearch Laboratories) in PBT for 18–36 h at 4 °C. Primary antibody incubations were conducted in blocking solution and incubated for 36–48 h at 4 °C. The tissues were then rinsed twice and washed 4× for 45 min. each in PBT. Secondary antibody, in blocking solution, were incubated for 36–48 h at 4 °C. Tissues were washed as described above for the primary antibody. Tissues were then cleared in 70% glycerol in PBS for 18–24 h at 4 °C. Tissues were mounted on slides in Vectashield (cat # H-1000–10, Vector Laboratories). Tissues were imaged using a Zeiss Axioscope epifluorescence microscope as well as a Zeiss LSM 510 and Leica SP5 confocal microscopes. Images were analysed using Fiji (Schindelin *et al.*, 2012). The following antibodies were used at the following concentrations: guinea pig anti-FOR (1:200, Belay *et al.*, 2007), mouse anti-Brp (1:50, Developmental Studies Hybridoma Bank), chicken anti-GFP (1:400, cat # A10262, Thermo Fisher Scientific), rabbit anti-GFP (1:200, cat # A-11122, Thermo Fisher Scientific), rat anti-mCherry (1:400, cat # M11217, Thermo Fisher Scientific), Alexa Fluor 546 Phalloidin (1:400, cat # A22283, Thermo Fisher Scientific), goat antichicken Alexa Fluor 488 (1:400, cat # A-11039, Thermo Fisher Scientific), goat antirabbit Alexa Fluor 488 (1:400, cat # A-11008, Thermo Fisher Scientific), goat antimouse Alexa Fluor 488 (1:400, cat # A-11001, Thermo Fisher Scientific), goat antimouse Alexa Fluor 633 (1:400, cat # A-21052, Thermo Fisher Scientific), goat anti-rat Alexa Fluor 546 (1:400, cat #A-11081, Thermo Fisher Scientific), goat antirat Alexa Fluor 633 (1:400, cat # A-21094, Thermo Fisher Scientific), goat antiguinea pig Alexa Fluor 633 (1:400, cat # A-21105, Thermo Fisher Scientific).

### Single-cell RNA-Seq data and analysis

The single-cell transcriptomic atlas of the adult brain was previously published (Davie *et al.*, 2018) and the data were acquired from Scope (http://scope.aertslab.org/#/e0816194-aea3-48d8-af80-569baf58be35/Aerts_Fly_AdultBrain_Filtered_57k.loom/gene). These cells were reprocessed as previously described (Allen *et al.*, 2020). Briefly, the data were processed in R (R Core Team, 2020) with Seurat v2.3.4 (Satija, Farrell, Gennert, Schier, & Regev, 2015). Data were normalized and scaled with ‘*NormalizData*’ and ‘*ScaleData*’ functions. A principal component analysis was run, with the ‘*RunPCA*’ function, on the variably expressed genes (as deduced by ‘*FindVariableGenes*’). A 2-dimensional t-distributed stochastic neighbor embedding (t-SNE; Van Der Maaten, Courville, Fergus, & Manning, 2014) was performed, using ‘*RunTSNE*’, on the first 82 principal components for visualization. Groups of cells with similar expression patterns were deduced with the ‘*FindClusters*’ function. The expression patterns of *repo* and *foraging* were visualized with the ‘*FeaturePlot*’ function. The per cluster average scaled expression was calculated with the ‘*AverageExpression*’ function and plotted using the pheatmap package (Kolde, 2019).

The single-cell transcriptomic atlas data of the adult midgut were previously published (Hung *et al.*, 2020) and the data were acquired from Gene Expression Omnibus (accession no. GSE120537). The data were processed with Seurat v3.2.2 (Stuart *et al.*, 2019) as described in the original publication (Hung *et al.*, 2020). Briefly, the data were normalized and scaled with ‘*NormalizData*’ and ‘*ScaleData*’ functions. Variably expressed genes were identified with the ‘*FindVariableGenes*’ function. The different replicates were integrated with the ‘*FindIntegrationAnchors*’ and ‘*IntegrateData*’ functions. A principal component analysis was run, with the ‘*RunPCA*’ function. A 2-dimensional Uniform Manifold Approximation and Projection (UMAP; McInnes, Healy, & Melville, 2018) was performed, using ‘*RunUMAP*’, for the first 30 principal components for visualization. Groups of cells with similar expression patterns were deduced with the ‘*FindClusters*’ function. The per cluster average scaled expression was calculated with the ‘*AverageExpression*’ function and plotted using the pheatmap package (Kolde, 2019).

## Results

### Foraging expression in the adult brain

**Figure 1.**
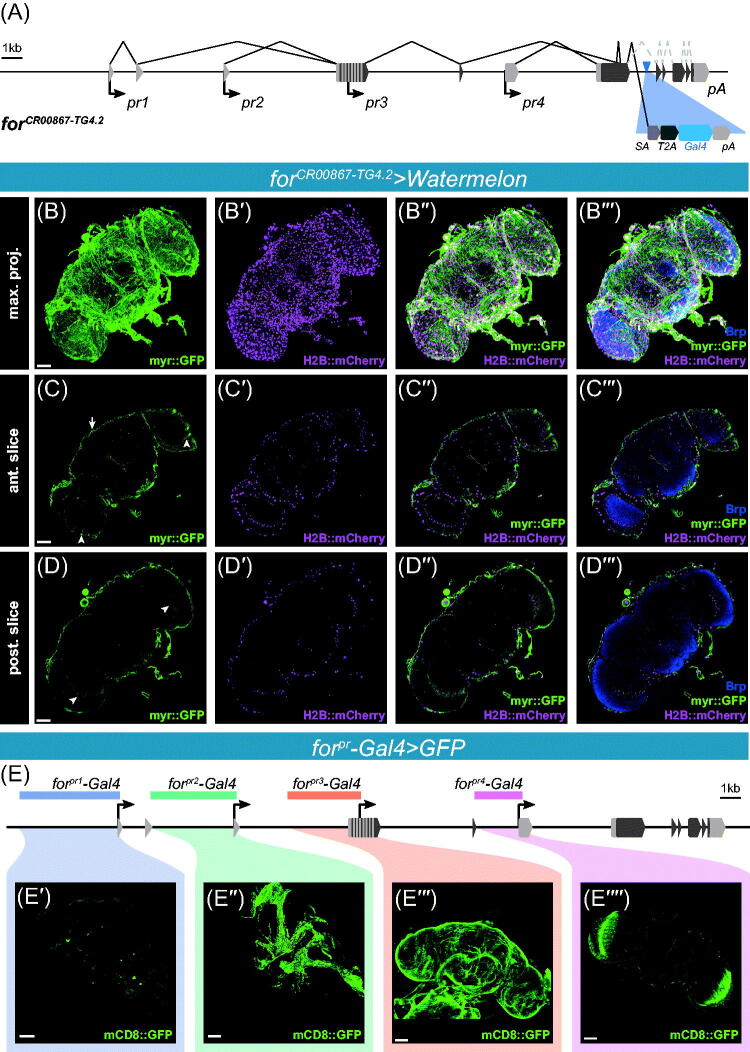
(A) Schematic of the *for^CR00867-TG4.2^* CRIMIC allele in the *foraging* locus. UTR regions are depicted with grey boxes and coding sequences are depicted with black boxes. Splicing patterns are depicted above the locus. The four transcription start sites are depicted below the locus, *pr1–4*. The CRIMIC element is inserted in the first intron after the first common coding exon (blue triangle). The splice acceptor sequence (SA) is designed to trap the endogenous transcription of *foraging*. The self-cleaving T2A sequence then allows for the translation of the *Gal4* coding sequence into a separate peptide. (pA – poly adenylation site). (B–B′′′) Maximal projections of the *for^CR00867-TG4.2^* CRIMIC allele driving *UAS-Watermelon* in the adult brain. Membrane bound GFP in green (B), nuclear mCherry in magenta (B′), membrane and nuclear merged (B′′), membrane and nuclear merged with Bruchpilot (nc82) in blue (B′′′). (C–C′′′) A single section in the anterior of the adult brain of the *for^CR00867-TG4.2^* CRIMIC allele driving *UAS-Watermelon*. Arrow denotes surface glia expression. Arrow heads in the optic lobes depict the cells with morphology consistent with outer chiasm glia. (D–D′′′) A single section in the posterior of the adult brain of the *for^CR00867-TG4.2^* CRIMIC allele driving *UAS-Watermelon*. Arrowheads in the optic lobes depict the cells with morphology consistent with inner chiasm glia. (E–E′′′′) Schematic of the *foraging* locus depicting regions of cloned *for^pr^-Gal4*s (E). *for^pr1^-Gal4* driven GFP expression in neurons innervating the optic lobe (E′). *for^pr2^-Gal4* driven expression in the trachea and air sacs (E′′). *for^pr3^-Gal4* driven expression in the perineurial surface glia (E′′′). *for^pr4^-Gal4* driven expression in the outer optic chiasm glia (E′′′′). Scale bars = 50 µm. [Please refer to the online version for colors.]

To infer the expression patterns and regulation of *foraging* in the adult, we took advantage of a novel *T2A*-based *Gal4* line and cloned promoter *Gal4* fusion lines from multiple regions of the locus. *T2A*-based *Gal4* alleles are designed to reliably capture endogenous gene expression due to being a transcriptional trap, as well as a translational trap. In the present study, we used the previously generated *for^CR00867-TG4.2^* CRIMIC allele (Lee *et al.*, 2018), which is integrated downstream of the first common coding exon (Figure 1(A)), allowing the splice acceptor sequence to capture the endogenous transcription from *foraging*. *for^CR00867-TG4.2^* is a homozygous lethal allele of *foraging*, dying as a pharate adult late in pupal development and does not complement the pupal lethality of the *for^0^* null allele (Allen *et al.*, 2017; Anreiter *et al.*, 2021). Heterozygous *for^CR00867-TG4.2^* driven *UAS-for^RNAi^* induces a consistent late pupal lethality (Anreiter *et al.*, 2021). We found that *for^CR00867-TG4.2^* drove expression in many cells with morphology consistent with multiple glial subtypes in the adult brain (Figure 1(B–D)). Most prominently, *for^CR00867-TG4.2^* expressed in the surface glia (Figure 1(C), arrow). Although we did not perform co-localization with a perineural marker, the surface glial pattern observed using the *for^CR00867-TG4.2^* was consistent with perineurial glia as evident from the number of cells (Figure 1(C′); Awasaki, Lai, Ito, & Lee, 2008). Previous studies found that *foraging* was among the top 50 most enriched genes when comparing surface glia transcriptomes to neuronal transcriptomes, and a protein trap allele *P{PTT-GB}for^CB02956^* also expressed in perineurial glia (DeSalvo *et al.*, 2014). Surface glia function as the blood–brain barrier in the larval and adult fly and regulate the transport of hormones, nutrients, and metabolites between the hemolymph and the brain (Bainton *et al.*, 2005; Laughlin, De Ruyter Van Steveninck, & Anderson, 1998; Limmer, Weiler, Volkenhoff, Babatz, & Klämbt, 2014; Schwabe, Bainton, Fetter, Heberlein, & Gaul, 2005; Stork *et al.*, 2008). Functions for glial subtypes and neuron–glia interactions in behavioral phenotypes have received relatively little attention (Artiushin & Sehgal, 2020; Bittern *et al.*, 2020).

Multiple distinct glial subtypes are found in the fly brain, all of which play distinct roles (Kremer, Jung, Batelli, Rubin, & Gaul, 2017; Yildirim, Petri, Kottmeier, & Klämbt, 2019). *for^CR00867-TG4.2^* drove expression in many of these subtypes. The next strongest expression in glial subtypes was found in the outer optic chiasm glia (Figure 1(C), arrowhead) and the inner optic chiasm glia (Figure 1(D), arrowhead). The outer optic chiasm glia have a fine, wispy structure found between the lamina and medulla (Kremer *et al.*, 2017). The inner chiasm glia fill the space between the medulla and the lobula and lobula plate (Kremer *et al.*, 2017). Weaker expression was seen in the neuropil- and tract-ensheathing glia (Figure S1(A,B)) and in the cortex glia (Figure S1(C–C′′)).

We also observed expression in the tracheal system surrounding and innervating the brain (data not shown). The *for^CR00867-TG4.2^* allele drove expression in both the large air sacs and the fine branching trachea in the brain. *foraging* expression in larval trachea has previously been found (Leader *et al.*, 2018).

To map the regulatory elements responsible for the observed expression, we next examined expression from the *for^pr^-Gal4* constructs (Figure 1(E)). The cloned regions encompassed 2–5kb upstream of each transcription start site (TSS) to 200 bp downstream of each TSS. These regions were cloned into a *Gal4* vector and inserted into the *attP2* landing site (described in Allen *et al.*, 2018). The *for^pr^-Gal4* regulatory regions together cover 15 kb of the 35 kb *foraging* locus. As was previously characterized in the larval CNS (Allen *et al.*, 2018), each *for^pr^-Gal4* line showed different expression patterns in the adult brain (Figure 1(E′–E′′′′)). In adults, *for^pr1^-Gal4* drove expression in only a few neurons in the brain (Figure 1(E′)). We saw the most robust expression in a pair of neurons with arbors connecting the suboesophageal zone to the medulla. None of these neurons were evident in the expression patterns observed with the *for^CR00867-TG4.2^* allele. *for^pr2^-Gal4* expressed in the trachea and air sacs surrounding and innervating into the adult brain, but there was no observable neuronal or glial expression (Figure 1(E′′)). *for^pr3^-Gal4* drove expression in the surface glia (Figure 1(E′′′), Figure S1(D)). This expression is consistent with perineurial glia as was seen in *for^CR00867-TG4.2^*. *for^pr4^-Gal4* expressed in the outer and inner chiasm glia of the optic lobes (Figure 1(E′′′′), Figure S1F).

The *for^pr-^Gal4*s do not express in all of the cells observed in *for^CR00867-TG4.2^* but mostly comprise a subset of the brain expression pattern observed in *for^CR00867-TG4.2^*. As less than half of the *foraging* locus is covered by these *for^pr–^Gal4*s lines, the necessary CREs for the remaining expression may lie in the un-cloned regions. Nevertheless, we found parallels between the expression patterns observed using the *for^CR00867-TG4.2^* gene trap and cloned *for^pr–^Gal4*s.

**Figure 2.**
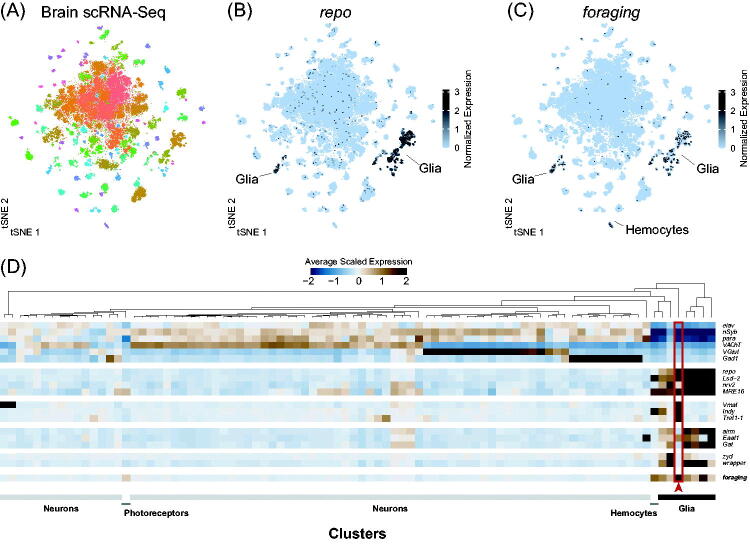
(A) t-SNE plot of single-cell RNA sequencing from the adult brain (data from Davie *et al.*, 2018). Each point represents the transcriptome of a single cell. Cells are clustered based on similarity of gene expression. Distinct cell types are represented by different colors. (B) Expression of the glial specific transcription factor *repo* in the single-cell brain atlas. *repo* expression is restricted to only a few clusters of cells. Cells are color coded according to the level of normalized expression. (C) Expression of *foraging* in the single cell brain atlas. *foraging* expression is restricted to a few clusters, most of which were also *repo* positive, and one was *Hml* positive. (D) Heatmap showing the average scaled expression of neuronal and glial marker genes across each cell cluster (colors in A). Neurons are marked by the pan-neuronal markers *elav*, *nSyb*, *para*, as well as the neurotransmitter specific genes *VAChT* (Acetylcholine), *VGlut* (Glutamate), and *Gad1* (GABA). Glia are marked by *repo*, *MRE16*, *nrv2*, *Lsd-2*. The genes *Vmat*, *Indy*, and *Tret1-1* label perineurial glia (among other things). *alrm*, *Gat*, and *Eaat1* label astrocyte-like glia. *zyd* labels cortex glia and ensheathing glia, and *wapper* labels tract cortex glia. *foraging* is enriched in all the glia clusters, as well as the *Hml* expressing hemocyte cluster. [Please refer to the online version for colors.]

In recent years, the advent of massively parallel single-cell transcriptomics has led to the unprecedented characterization of gene expression of individual cell types. Multiple data sets characterize the transcriptomic profiles of different subsets of the adult CNS (Allen *et al.*, 2020; Croset, Treiber, & Waddell, 2018; Davie *et al.*, 2018; Gao *et al.*, 2015; Konstantinides *et al.*, 2018; Li *et al.*, 2017). We took advantage of a whole brain data set (Davie *et al.*, 2018) to explore *foraging* expression at the single-cell resolution. Individual cells are grouped based on the similarity of their gene expression profiles, and different colors represent distinct cell types (see “Methods”, Figure 2(A)). Using the established glial marker gene *repo*, we can identify the glial clusters (Figure 2(B)). *foraging* expression was for the most part restricted to *repo* positive clusters, but expression was also seen in hemocytes and photoreceptors (Figure 2(C,D)). Looking at the average scaled expression for multiple neuronal, glial, and glial sub-type markers across each cluster of cells (colors in Figure 2(A)), it is clear that *foraging* is specifically enriched in glia with highest enrichment in perineurial glia (Figure 2(D), red arrow).

The *for^CR00867-TG4.2^* allele (Figure 1(B–D)) and the single-cell transcriptomics data (Figure 2(A–D)) found no detectable neuronal expression in the adult fly brain. We were surprised not to detect neuronal expression in the adult fly brain because functional studies that manipulate *foraging* levels in neurons are known to alter several behavioral phenotypes (reviewed in Anreiter and Sokolowski 2019 and discussed further below). Consequently, we used two more *Gal4* alleles, the *for^MI01791-TG4.1^* (Diao *et al.*, 2015; Lee *et al.*, 2018) and *BAC{for^IRES-Gal4^}* to investigate *foraging* expression in the adult brain. The *for^MI01791-TG4.1^* allele also drove expression in the surface glia, and similar to the CRIMIC allele did not have any detectable neuronal expression (Figure S1(G–G′′)). Both the *for^MI01791-TG4.1^* and *for^CR00867-TG4.2^* alleles rely on a splice acceptor and *T2A* sequence to trap the endogenous transcription and translation of *foraging*. We also implemented an independent strategy to generate a recombineered bacterial artificial chromosome (BAC), containing the entire *foraging* locus, with the coding sequence common to all isoforms replaced with an *Internal-Ribosomal-Entry-Sequence* and *Gal4* coding sequence. An un-mutated copy of this BAC was previously shown to have similar expression, as measured by RT-qPCR and western blot, to that of wild-type flies, and was sufficient to rescue the *for^0^* null mutant in many of *foraging*’s larval associated phenotypes (Allen *et al.*, 2017; Dason *et al.*, 2019). This *BAC{for^IRES-Gal4^}* allele drove expression in the surface glia and again had no detectable neuronal expression (Figure S1(H–H′′)). We provide a more detailed expression analysis using the *for^CR00867-TG4.2^* allele as its expression was stronger than the *for^MI01791-TG4.1^* and *BAC{for^IRES-Gal4^}* alleles.

**Figure 3.**
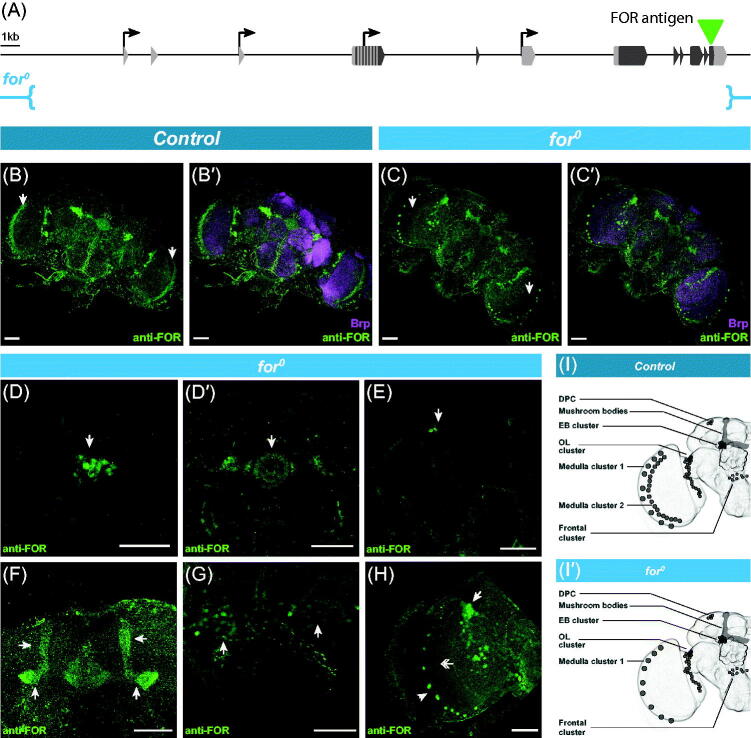
(A) Schematic of the *foraging* gene (as in Figure 1(A)). The 120bp (40aa) antigenic region used to generate the polyclonal anti-FOR antibody is depicted with a green arrowhead (described in Belay *et al.*, 2007). The *for^0^* genetic deletion (described in Allen *et al.*, 2017) and its break points (in blue) are depicted below. (B) Maximal projection of anti-FOR antibody staining in control animals of late pupal brains (4 days post-puparium formation). Arrows in optic lobes indicating cells with morphology consistent with outer optic chiasm glia. Neuropil visualised with anti-Brp (B′). (C) Maximal projection of anti-FOR antibody staining in *for^0^* null mutant (described in Allen *et al.*, 2017) animals of late pupal brains (4 days post-puparium formation). Arrows in optic lobes indicating lack of expression in cells with morphology consistent with outer optic chiasm glia. Neuropil visualized with anti-Brp (C′). (D–H) Magnification of anti-FOR positive clusters in the *for^0^* null mutant. Immunoreactivity in the ellipsoid body cluster, EB cluster (arrow, D) and its projections into the ellipsoid body (arrow, D′). Staining was also seen in the 4 cells of the dorsal posterior cluster, DPC (arrow, E). Staining in the mushroom bodies (arrow, F). Staining in the frontal cluster (arrow, G). Staining in the optic lobe cluster, OL cluster (arrow, H), medulla cluster 1 (arrowhead, H), and absent in medulla cluster 2 (double arrow, H). (I–I′) Schematics of anti-FOR expression patterns in control and *for^0^* null mutant animals. Expression in the *for^0^* null mutant was the same as control except for the lack of medulla cluster 2. Scale bars = 50 µm. [Please refer to the online version for colors.]

Previous studies characterized the expression of *foraging* proteins in the adult brain using an anti-FOR antibody (Belay *et al.*, 2007; Mery *et al.*, 2007). A 40 amino acid sequence from the C-terminus shared by all predicted FOR isoforms was used to generate the polyclonal antibody used in this study (Figure 3(A)). Alignments showed that the full C-terminal segment used to make this antibody was not encoded by any other sequences in the *D. melanogaster* genome (Belay *et al.*, 2007). At the time of publishing Belay *et al.* (2007), we did not possess a complete genetic deletion of *foraging*. Consequently, we used a partial deletion of *foraging* called *Df(2L)ED243* to confirm the effectiveness of this anti-FOR antibody. This deletion removed 2 of the 4 TSSs of *foraging* spanning from 50 bp downstream of *for^pr1^* to 65 bp upstream of *for^pr4^* (Ryner *et al.*, 1996). In Belay *et al.* (2007), the specificity of the anti-FOR antibody in immunohistochemical analyses was evaluated using the larval proventriculus instead of adult tissue because homozygous *Df(2L)ED243* do not survive to adulthood. Immunoreactivity in the larval proventriculus was absent in *Df(2L)ED243* homozygous larvae compared to the control. Localized signal in the adult brain was also missing when tissues were incubated with the preabsorbed antibody. Belay *et al.* (2007) concluded that the anti-FOR antiserum detects endogenous FOR.

However, we recently published a full 35 kb genetic null of the *foraging* gene, *for^0^* (Allen *et al.*, 2017). The stage of lethality of the *for^0^* null mutant is late pupal lethal (Allen *et al.*, 2017; Anreiter *et al.*, 2021). This new allele allowed us to re-examine the efficacy of the anti-FOR staining using late stage *for^0^* null mutant pupal brains (4 days post-pupariation). Anti-FOR antibody staining in control pupae showed the same expression patterns previously reported in adult heads (Figure 3(B); Belay *et al.*, 2007; Mery *et al.*, 2007). All of the previously reported primary anti-FOR clusters were seen in the pupal brain, optic lobe cluster, medulla cluster, ellipsoid body cluster, frontal cluster, dorsal posterior cluster, and the mushroom bodies. However, when we examined the *for^0^* pupal brains, all of these clusters remained (Figure 3(C)), except for part of the medulla cluster (Figure 3(B,C), arrows). Specifically, immunoreactivity in the *for^0^* null brains was seen in the ellipsoid body cluster (Figure 3(D–D′)), the dorsal posterior cluster (Figure 3(E)), the mushroom bodies (Figure 3(F)), the frontal cluster (Figure 3(G)), the optic lobe cluster (Figure 3(H), arrow), and medulla cluster 1 (Figure 3(H), arrowhead), but was lacking from medulla cluster 2 (Figure 3(H), double-headed arrow). The medulla cluster 2 was previously reported to be ELAV negative (Belay *et al.*, 2007), has a similar characteristic structure to that of the outer optic chiasm glia (Kremer *et al.*, 2017), and is consistent with that seen in the *for^CR00867-TG4.2^* and *for^pr4^-Gal4* expression patterns (Figure 1(C,E′′′′)). This finding suggests that in the adult brain, the anti-FOR antibody labels *bone fide* FOR expression in the outer optic chiasm glia but also has significant non-specific immunoreactivity suggesting that the majority of the previously described primary expression patterns in the adult brain is not FOR. Previous functional studies that showed significant effects on adult behaviors when manipulating *foraging* expression in the mushroom bodies and ellipsoid body are discussed below.

It is notable that the anti-FOR antibody displays high specificity to FOR proteins in western blot analyses of control and *for^0^* mutant larvae and that the *for^0^* null mutants did not display any immunoreactivity (Allen *et al.*, 2017; Dason *et al.*, 2019). We confirmed that there were no strong matches other than FOR along the whole 40 amino acid sequence (Belay *et al.*, 2007), with a BLASTp search. However, low-affinity targets may be of concern when non-specificity of an antibody is found (Fritschy, 2008), and we did find smaller stretches of significant similarity with Protein kinase A catalytic subunits Pka-C1 and Pka-C3 (data not shown). Both Pka-C1 and Pka-C3 have been shown to be expressed in the Kenyon cells of mushroom bodies (Croset *et al.*, 2018; Davie *et al.*, 2018; Davis *et al.*, 2020; Skoulakis, Kalderon, & Davis, 1993). Further purification of this antibody may ameliorate its performance in the context of whole mount immunohistochemistry, for instance, pre-absorbing the antibody with protein extracted from *for^0^* mutants. The anti-FOR antibody specificity and utility works well to detect FOR proteins on westerns but not in immunohistochemical analysis. The context specificity of the utility of antibodies is a common problem (Baker, 2015).

### Foraging expression in the adult gastric system

**Figure 4.**
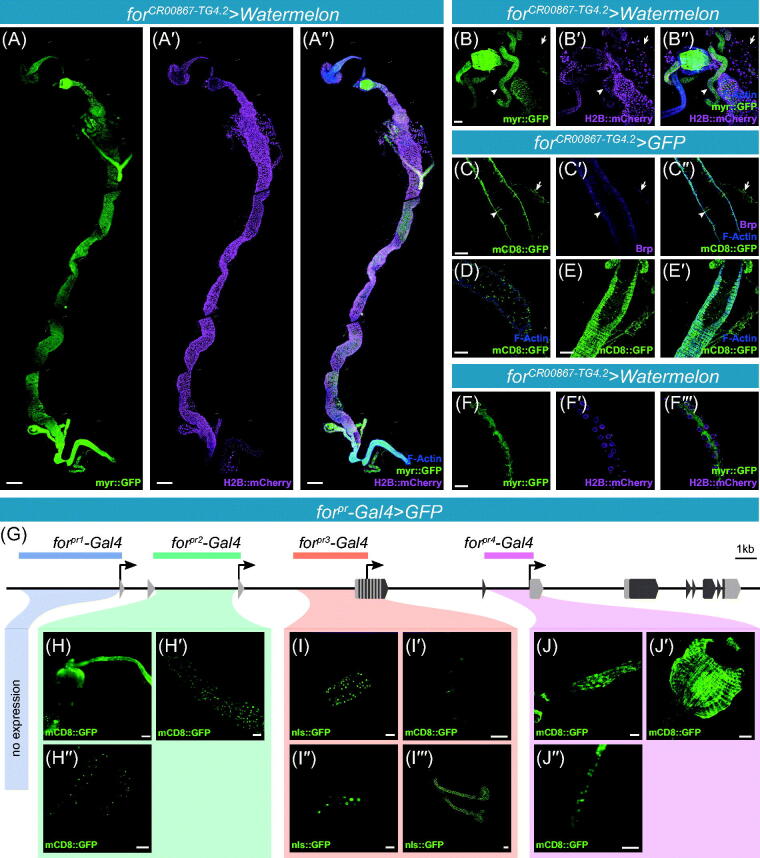
(-A″) Maximal projections of the *for^CR00867-TG4.2^* CRIMIC allele driving *UAS-Watermelon* in the adult gut. Membrane bound GFP in green (A), nuclear mCherry in magenta (A′), membrane and nuclear merged with F-actin (A′′). Scale bar = 200 µm. (B–B′′) Maximal projections of *for^CR00867-TG4.2^* driving *UAS-Watermelon* in the cardia and other tissues. Membrane bound GFP in green (B), nuclear mCherry in magenta (B′), membrane and nuclear merged with F-actin in blue (B′′). White arrow heads indicating the salivary gland. White arrows indicating the Malpighian tubules. (C–C′′′) Single section of *for^CR00867-TG4.2^* driving *UAS-mCD8::GFP* in the anterior midgut. GFP expression can be seen in the enteroendocrine (EE) cells and the muscle (C). EE cells can be identified by the expression of Brp (C′), and muscles with F-actin (C′′). White arrowhead indicating an example of co-expression of GFP and Brp (C′′′). (D) Single section of *for^CR00867-TG4.2^* driving *UAS-mCD8::GFP* in the intestinal stem cells of the midgut. (E–E′) Subsection of *for^CR00867-TG4.2^* driving *UAS-mCD8::GFP* in the visceral muscle of the midgut. (F–F′′′) Maximal projections of *for^CR00867-TG4.2^* driving *UAS-Watermelon* in the Malpighian tubules. (G) Schematic of the of the *foraging* locus depicting regions of cloned *for^pr^-Gal4*s. (H–H′′) *for^pr2^-Gal4* driven expression in the gastric system. Expression was seen in the foregut and cardia (G), as well as the midgut intestinal stem cells (G′), and the Malpighian stem cells in the ureter (G′′). I–I′′′. *for^pr3^-Gal4* driven expression in the gastric system. Expression was seen in middle midgut enterocytes (H), anterior EE cells (H′), distal segment of the Malpighian tubules (H′′), and the salivary glands (H′′′). J–J′. *for^pr4^-Gal4* driven expression in the gastric system. Expression was seen in epithelia of the hindgut (I), the ampulla (I′), and the salivary duct (I′′). B–J. Scale bars = 50 µm. [Please refer to the online version for colors.]

The gastric system plays a crucial and central role in feeding-related phenotypes. Not only is it essential for the digestion of food, absorption of nutrients, and excretion of waste, but also it provides feedback to the brain via hormone signaling that can affect behavior (reviewed in Miguel-Aliaga, Jasper, & Lemaitre, 2018). The *foraging* gene influences many feeding-related phenotypes suggesting that *foraging* may have functions in the adult gastric system. We found that the *for^CR00867-TG4.2^* allele expressed throughout the gastric system (Figure 4(A–F)). Expression was evident in all major compartments (foregut and crop, cardia, midgut, hindgut, and Malpighian tubules) of the gastric system and all major cell types.

Expression was found in the esophagus, foregut (crop duct), and crop (Figure 4(A,B)). The crop provides a central role in nutrient sensing and digestion in the adult fly (Hadjieconomou *et al.*, 2020). *for^CR00867-TG4.2^* expressed in the enterocytes (ECs; Figure 4(A,B)) and enteroendocrine cells (EE cells; Figure 4(C)) of the midgut. The EE cells can be identified by their morphology and by co-expression with Brp (Figure 4(C′); Zeng, Lin, & Hou, 2013). The ECs and EEs are important for absorption and digestion of ingested nutrients. Moving from the anterior to the posterior, digestion shifts from larger macromolecules to smaller monosaccharides. We also observed expression in the intestinal stem cells of the midgut (Figure 4(D)). The ISCs are important for midgut homeostasis (Micchelli & Perrimon, 2006; Ohlstein & Spradling, 2006; reviewed in Miguel-Aliaga *et al.*, 2018).

Expression was found in the visceral muscle throughout the entire length of the gut, from the foregut to the hindgut (Figure 4(E)). The visceral muscle is required to push these nutrients along via peristalsis. *for^CR00867-TG4.2^* was expressed throughout the Malpighian tubules (Figure 4(B), arrow; Figure 4(F)). The tubules are vital for ion balance in the fly's hemolymph. There was strong expression in the hindgut proliferation zone of the pylorus, and in the epithelial cells throughout the rest of the hindgut and ampulla (Figure 4(A)). Much of the hindgut is important for ion absorption, and the ampulla is crucial for water balance (Lemaitre & Miguel-Aliaga, 2013). *for^CR00867-TG4.2^* also drove expression in the salivary glands (Figure 4(B), arrowhead) and the trachea innervating the gut (Figure 4(C), arrow).

We next turned to our *for^pr^-Gal4* driver lines to parse this expression pattern and map its regulatory elements (Figure 4(G)). As for the larva, we found a modular pattern of expression in the adult. We found no detectable expression from *for^pr1^-Gal4* in the gastric system. *for^pr2^-Gal4* expression was found in the epithelia of the foregut, crop, and cardia (Figure 4(H)). *for^pr2^-Gal4* expression was also seen in the midgut ISCs (Figure 4(H′)) as typified by co-expression with DELTA (Figure S2(A); Micchelli & Perrimon, 2006; Ohlstein & Spradling, 2006 ) and in the EE precursor cells (pre-EE) as typified by co-expression with PROSPERO (Figure S2(B); Zeng & Hou, 2015). *for^pr2^-Gal4* expression was found in the stem cell zone of the ureter and lower Malpighian tubule (Figure 4(H′′); Singh & Hou, 2009; Sözen, Armstrong, Yang, Kaiser, & Dow, 1997; Wang & Spradling, 2020). The *for^pr3^-Gal4* drove expression in a narrow band of ECs in the mid-region of the midgut, a few anterior EEs, visceral gut muscle, and the principal cells of the transitional segment of the Malpighian tubules (Figure 4(I–I′′)). *for^pr4^-Gal4* drove expression in the epithelia of the hindgut and in the rectal ampulla (Figure 4(J–J′)). *for^pr3^-Gal4* drove expression throughout the salivary glands (Figure 4(I′′′)), and *for^pr4^-Gal4* was restricted to the salivary duct of the salivary gland (Figure 4(J′′)). Overall, the expression seen in the *for^pr^-Gal4* lines is a subset of the expression found using the *for^CR00867-TG4.2^* allele.

**Figure 5.**
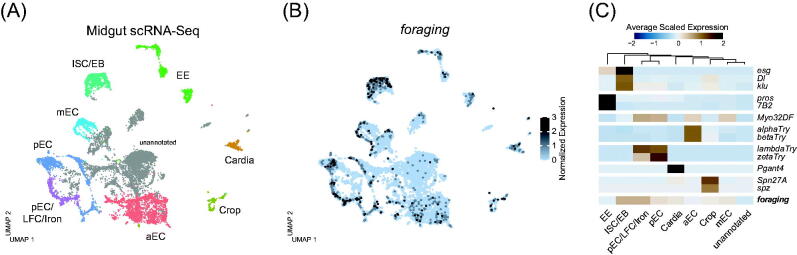
(A) UMAP plot of single-cell RNA sequencing from the adult midgut (data from Hung *et al.*, 2020). Each point represents the transcriptome of a single cell. Cells are clustered based on similarity of gene expression. Distinct cell types are represented by different colors. EE: enteroendocrine cells; ISC: intestinal stem cells; EB: enteroblasts; LFC: large flat cells; Iron: iron cells; aEC: anterior enterocytes; mEC: middle enterocytes; pEC: posterion enterocytes. (B) Expression of *foraging* in the single-cell midgut Atlas. Cells are color coded according to the level of normalized expression. (C) Heatmap showing the average scaled expression of cell type marker genes across each cell cluster. Initialisms are defined in A. *foraging* is most enriched in the ISC/EB and pEC/LFC/Iron clusters. EE cells are marked by *pros* and *7B2*. ISC/EB are marked by *esg*, *Dl*, and *klu*. All ECs are marked by *Myo32DF*, and the anterior to posterior access is delineated by a series of trypsin coding genes (*alphaTry*, *betaTry*, *lambdaTry*, *zetaTry*, among others). The cardia is marked by *Pgant4*, and the crop by *Spn27A* and *spz*. [Please refer to the online version for colors.]

*foraging* transcripts have previously been detected throughout the gastric system in dissected bulk RNA-Seq experiments (Buchon *et al.*, 2013; Leader *et al.*, 2018), and sorted cell types from the midgut (Dutta *et al.*, 2015). *foraging* transcripts were detected in all major cell types (ISC, EB, EE, EC, and VM) in all major segments (cardia and R1, R2, R3, R4, and R5) of the midgut (Dutta *et al.*, 2015). To further explore and validate *foraging's* expression in the gastric system, we turned to the adult midgut's single-cell transcriptomic atlas (Figure 5(A); Hung *et al.*, 2020). Cell types were identified using known and established markers (as described in Hung *et al.*, 2020). *foraging* expression was seen in all the midgut cell types (Figure 5(B)), with its expression most enriched in the ISC/EB cluster (Figure 5(C)).

### Foraging expression in the adult reproductive systems

**Figure 6.**
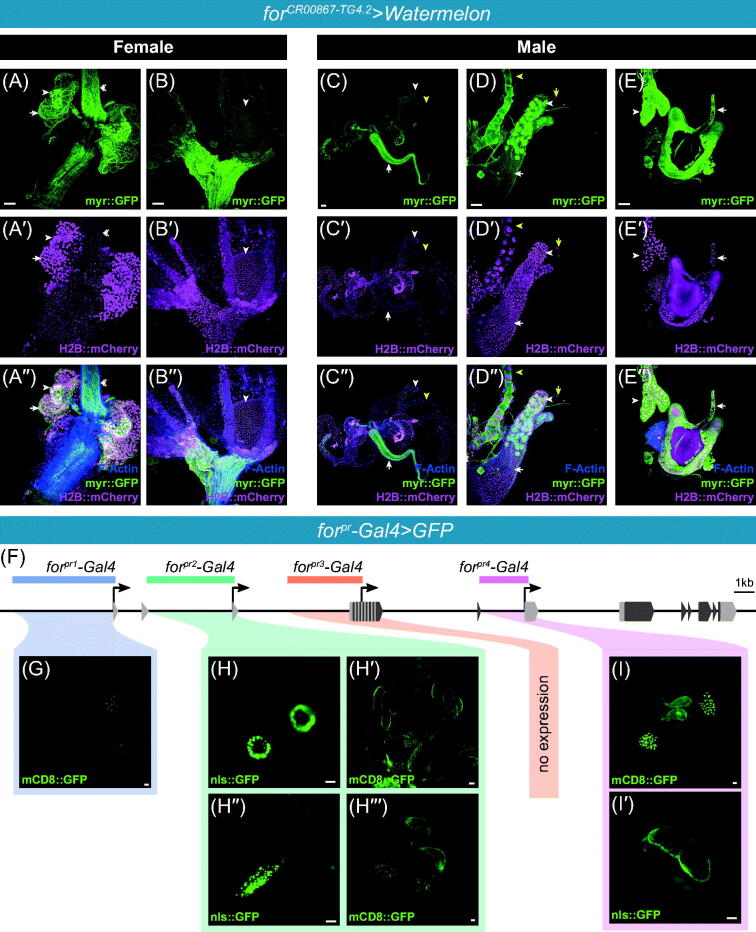
**(**A–E′′′) *for^CR00867-TG4.2^* CRIMIC allele driving *UAS-Watermelon* in female and male reproductive systems with membrane bound GFP in green (A–E), nuclear mCherry in magenta (A′–E′), membrane and nuclear merged with F-actin in blue (A′′–E′′). (A–A′′) Maximal projections of expression in the female reproductive system; uterus, spermatheca (white arrowhead), and common oviduct (white double arrowhead) of the female reproductive system. Expression was also seen in the spermatheca associated fat (white arrow) and smooth muscle. (B–B′′) Maximal projections of expression in the female reproductive system; ovarioles and lateral oviducts. Expression was seen in the common and lateral oviducts, epithelial sheath surrounding the ovariole, and follicle cells (arrowhead). (C–C′′) Maximal projections of expression in the male reproductive system. Expression was seen throughout; ejaculatory duct (white arrow), seminal vesicles, testis, and accessory glands (white arrowhead). Other tissues are also present (Malpighian tubules – yellow arrowhead). (D–D′′) Magnification of the accessory gland. Primary cells indicated with white arrow, and secondary cells indicated with white arrowhead. Other tissues are also present (Malpighian tubules – yellow arrowhead, trachea – yellow arrow). (E–E′′) Magnification of the ejaculatory bulb. Lower ejaculatory duct (white arrow) and fat tissue (white arrowhead) are indicated. (F) Schematic of the *foraging* locus depicting regions of cloned *for^pr^-Gal4*s. G. *for^pr1^-Gal4* driven GFP expression in the male reproductive system. (H) *for^pr2^-Gal4* driven expression in the spermatheca. (H′) *for^pr2^-Gal4* driven expression in the ovaries. H′′. *for^pr2^-Gal4* driven expression in the oviduct. (H′′′) *for^pr2^-Gal4* driven expression in the male reproductive system. (I) *for^pr4^-Gal4* driven expression in the male reproductive system. (I′) *for^pr4^-Gal4* driven expression in the ejaculatory bulb. Scale bars = 50 µm. [Please refer to the online version for colors.]

*for^CR00867-TG4.2^* was expressed in most cell types throughout the female and male reproductive system. Expression was seen in the epithelia of the uterus, common oviduct, lateral oviducts (Figure 6(A,B)). Expression was observed in the ovarioles and epithelial sheath surrounding the ovarioles (Figure 6(B)). The most robust expression was seen in the spermatheca (Figure 6(A), arrowhead; Figure S2(C)) and fat cells associated with the spermatheca (Figure 6(A), arrow). *foraging* is also known to have high levels of expression in the spermatheca, from microarray and RNA-Seq experiments on dissected tissue (Chintapalli, Wang, & Dow, 2007; Leader *et al.*, 2018). *for^CR00867-TG4.2^* is also expressed in the follicle cells of the developing eggs (Figure 6(B), arrowhead). The maternal loading of *foraging* in the developing eggs and the early embryo has been well characterized (Graveley *et al.*, 2011; Jambor *et al.*, 2015; Koenecke *et al.*, 2016; Tomancak *et al.*, 2002).

*for^CR00867-TG4.2^* is expressed throughout the male reproductive system, with strongest expression in the anterior ejaculatory duct (Figure 6(C), white arrow), secondary cells of the accessory gland (Figure 6(C,D), white arrowhead), and ejaculatory bulb (Figure 6(E)). The male accessory glands produce many proteins required for fertility, and their secretions have been shown to have substantial effects on female post-mating behaviors (Wolfner, 1997). The ejaculatory bulb functions to pump the ejaculate and contributes to glandular secretions. Extensive expression was seen in the rest of the reproductive system, including the testes, seminal vesicle, and primary cells of the accessory gland (Figure 6(C,D)). The seminal vesicles are primarily a storage organ for mature sperm prior to copulation, and they also produce glandular secretions for the seminal fluid (Ram & Wolfner, 2007). Expression was also seen in the smooth muscle throughout the female and male reproductive systems.

Once again, we return to the *for^pr^-Gal4* lines to parse these expression patterns and map its regulatory elements. We saw expression of *for^pr2^-Gal4,* but not *for^pr1^-*, *for^pr3^-*, and *for^pr4^-Gal4,* in the female reproductive system. *for^pr2^-Gal4* expressed in the spermatheca (Figure 6(H)) and the follicle cells of the developing eggs (Figure 6(H′)). *for^pr2^-Gal4* also expressed in a small segment of the common oviduct (Figure 6(H′′)).

*for^pr1^-*, *for^pr2^-*, and *for^pr4^-Gal4* all drove expression in the seminal vesicle and secondary cells of the accessory glands, albeit to varying extents (Figures 6(H′′′, I, G), respectively). *for^pr2^-Gal4* had the added inclusion of a small ring of cells at the base of the vas deferens where it joins with the ejaculatory duct (data not shown). *for^pr4^-Gal4* drove expression in the ejaculatory bulb of the male reproductive system (Figure 6(I′)). *for^pr3^-Gal4* had no observed expression in the male reproductive system.

### *Mapping foraging*’s *cis*-regulatory elements (CREs)

**Figure 7.**
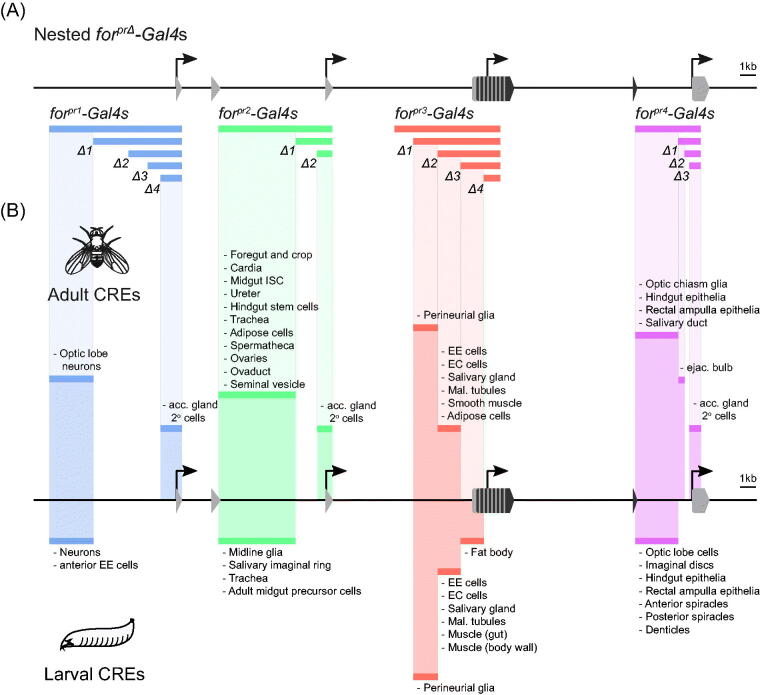
**(**A). Schematic of cloned regions for the nested *for^pr^-Gal4*s. The 5′-end of the *foraging* locus is depicted with regions corresponding to the 4 original *for^pr^-Gal4*s, and the 13 newly generated nested *for^prΔ^-Gal4*s are below the locus, each numbered according to ranked size (e.g. *for^pr1Δ1^-Gal4*, *for^pr1Δ2^-Gal4*, etc.). These regions were cloned into a *gypsy* insulated *Gal4* vector and inserted into *attP2* landing site with φC31 integration. (B) Mapped *cis*-regulatory elements (CREs) in the adult (depicted above the locus) and larval (depicted below the locus) *D. melanogaster*. CREs were mapped along the locus by comparing the expression patterns of the 17 nested cloned *for^pr^-Gal4*s.

To further map and refine the CREs within the *foraging* gene, we generated a series of 13 nested *for^prΔ^-Gal4* driver lines covering regions within the four original *for^pr^-Gal4*s (Figure 7(A), bars below locus). These regions were cloned into the same insulated vector used for the original *for^pr^-Gal4*s and were integrated into *attP2* with φC31 integration (see “Methods”). By comparing the expression patterns seen between these different lines, we could narrow the putative CREs in the *foraging* locus (Figure 7(B), above the locus). For instance, in the adult fly, *for^pr3^-Gal4* and *for^pr3Δ1^-Gal4* did not differ in their expression patterns, so we did not map any element to the first *for^pr3^* region. In contrast, *for^pr3Δ2^-Gal4* had all the expression of *for^pr3Δ1^-Gal4*, except for the perineurial glia expression. This allowed us to map the perineurial CRE to this second segment of the *for^pr3^* region. All nested *for^prΔ^-Gal4*s exhibited either the same or a subset of the expression patterns seen in their respective larger constructs.

Many genes have highly conserved promoter structure and show consistent expression across development (Graveley *et al.*, 2011; Hoskins *et al.*, 2011). Is this trend also true for *foraging?* The *for^pr1-4^-Gal4* lines were previously characterized for their expression in the larval CNS and gastric system (Allen *et al.*, 2018). Examining the expression of the newly generated nested lines allows us to compare the regulation between the larva and adult fly. As was seen in the adult, the expression in the larvae of the nested *Gal4* lines were either the same or a subset of the expression seen in the larger *for^pr^-Gal4* lines. A number of these refined CREs drove comparable expression in the adult (Figure 7(B), above the locus) and the larvae (Figure 7(B), below the locus). For example, the first region from *for^pr1^-Gal4* drove expression in neurons. The first region from *for^pr2^-Gal4* drove expression in the larval and adult intestinal stem cells (“Adult midgut precursor cells” and “Midgut ISC”, respectively). The perineurial CRE and the CRE driving expression in the salivary gland, EE cells, ECs, and Malpighian tubules mapped in the *for^pr3^-Gal4*s also drove consistent expression in the larva and adult. The CRE driving expression in the epithelia of the hindgut and rectal ampulla from *for^pr4^-Gal4*s was also shared. These data provide evidence that *foraging* has conserved promoter structure exhibiting consistencies in expression between the larval and adult stage of development.

## Discussion

Relatively little is known about the precise spatial- and cell-specific expression of *foraging,* a highly conserved gene with a complex gene structure (reviewed in Anreiter & Sokolowski, 2019). Here we set out to characterize *foraging* gene expression in the adult brain, gastric system, and reproductive systems of *D. melanogaster*. We employed a multimodal approach by combining *foraging* gene-trap *T2A-Gal4*, promoter *Gal4* fusions, and the mining of existing single-cell transcriptomic data sets. The advantages and limitations of these techniques are described below.

Trojan/CRIMIC style insertion alleles are unparalleled in their expression fidelity due to their function as both a transcriptional and translational trap of the locus (Diao *et al.*, 2015; Lee *et al.*, 2018). Unlike insertion alleles made with older technologies, Trojan/CRIMIC insertion alleles significantly limit offsite effects on the readout of a gene’s expression. Consequently, it is more likely that the *for^CR00867-TG4.2^* allele, inserted into the *foraging* locus, captures the expression of all known *foraging* isoforms and therefore, should represent the full *foraging* expression pattern (Figure 1(A)).

Promoter fusion reporters have been widely used to characterize the spatial expression pattern of genes in *D. melanogaster*. However, the position where they are integrated into the fly’s genome can significantly alter the level and patterns of expression that are seen (Kellum & Schedl, 1991; Markstein, Pitsouli, Villalta, Celniker, & Perrimon, 2008; Spradling & Rubin, 1983). Problems with position effects can be reduced using *gypsy* insulators (Barolo *et al.*, 2000; Billeter & Goodwin, 2004; Gdula, Gerasimova, & Corces, 1996; Markstein *et al.*, 2008) and φC31 site-specific integration (Groth *et al.*, 2004; Markstein *et al.*, 2008). We employed both these strategies when constructing our *for^pr^-Gal4* transgenic constructs. The lack of shared expression between the largest of each *for^pr^-Gal4* constructs suggests a lack of position effects from our *for^pr^-Gal4* insertions or expression driven by the empty *Gal4* vector itself. An enhancer’s ability to induce expression can also be dependent on the core promoter element it is paired with (Lehman *et al.*, 1999; Merli, Bergstrom, Cygan, & Blackman, 1996; Ohtsuki, Levine, & Cai, 1998). Our lines exploited *foraging*’s native core promoters and TSS from the locus. Together, these strategies should increase the likelihood that the expression we found in our *for^pr^-Gal4* experiments faithfully recapitulates *foraging* expression.

Massively parallel single-cell transcriptomic experiments provide unprecedented cellular resolution of gene expression but are not without their caveats. For instance, droplet-based methods suffer from a number of factors, such as drop-out events where only a subset of the transcriptome from a cell is captured (Kim, Zhou, & Chen, 2020; Qiu, 2020), doublet and multiple formation where multiple cells are co-encapsulated (Bernstein *et al.*, 2020; McGinnis, Murrow, & Gartner, 2019; Wolock, Lopez, & Klein, 2019), and contamination of ambient RNA generated during the dissection and dissociation (Yang *et al.*, 2020; Young & Behjati, 2018). Sufficient sampling of each given cell type along with the above cited bioinformatic approaches can reduce these issues.

Overall, we found *foraging* expressed in multiple glia sub-types in the brain with the strongest expression seen in the perineurial glia. This was supported by the *T2A-Gal4* alleles *for^CR00867-TG4.2^* (Figure 1(B–D)) and *for^MI01791-TG4.1^* (Figure S1(G)), the *BAC{for^IRES-Gal4^}* recombineered allele (Figure S1(H)), the *for^pr3^-Gal4* cloned promoter *Gal4* transgene (Figure 1(E′′′); Figure S1(D–E)), single-cell RNA-Seq data sets (Figure 2(A–D); Davie *et al.*, 2018; Croset *et al.*, 2018), and bulk RNA-Seq data on sorted cells (DeSalvo *et al.*, 2014). No clear neuronal expression was seen in the brain in either gene-trap allele, or the single-cell transcriptomic data, however, a few neurons were labelled with *for^pr1^-Gal4* (Figure 1(E′)). We also show that the previously characterized anti-FOR immunoreactivity in adult brain neurons (Belay *et al.*, 2007; Mery *et al.*, 2007) is non-specific and does not reflect *bone fide* FOR expression (Figure 3). Finally, we also found that the *foraging* gene expressed extensively throughout the gastric and reproductive systems (Figures 4–6) and the expression in these tissues and cells was corroborated with data from microarray, bulk RNA-Seq, and single-cell RNA-Seq based transcriptomic studies (Brown *et al.*, 2014; Buchon *et al.*, 2013; Chintapalli *et al.*, 2007; Dutta *et al.*, 2015; Hung *et al.*, 2020; Leader *et al.*, 2018). Each of these results are discussed in more detail below.

### Foraging in the adult brain

Glia are intwined with neuronal physiology and behavior. Glia influence the development and function of the synapse, neurotransmitter release, ion homeostasis and immune function throughout an organism’s life (reviewed in Artiushin & Sehgal, 2020). The number of studies of the role of glia in behavior is growing rapidly but is still much more limited than their neuronal counterparts. Glia serve important roles in behavior (reviewed in Freeman, 2015; Zwarts, Van Eijs, & Callaerts, 2015). For example, in *D. melanogaster*, glia function in embryonic motility and adult locomotion (Lehmann & Cierotzki, 2010; Pereanu *et al.*, 2007) and in male courtship behavior (Grosjean, Grillet, Augustin, Ferveur, & Featherstone, 2008). A subset of glia called astrocytes function in sleep and circadian rhythms of locomotion and their cellular correlates (Artiushin & Sehgal, 2020).

At the larval neuromuscular junction (NMJ) glial-specific expression of *foraging* decreases neurotransmitter release by negatively regulating nerve terminal growth (Dason *et al.*, 2019; Dason & Sokolowski, 2021). The *for^0^* null mutant has increased nerve terminal growth which can be rescued by glial-specific, and not neuronal-specific (pre-synaptic) or muscle-specific (post-synaptic) expression of *foraging* (Dason *et al.*, 2019). Furthermore, glial-specific knockdown of *foraging* phenocopies the nerve terminal over-growth and increased neurotransmitter release phenotypes seen in the *for^0^* null mutant (Dason & Sokolowski, 2021). Knockdown of *foraging* in presynaptic neurons of the NMJ impairs synaptic vesicle endocytosis, whereas knockdown of *foraging* in glia does not. Overall, *D. melanogaster foraging* can alter neurotransmitter release at the NMJ by regulating both synaptic structure and function. Similar functions are likely also at play in the adult nervous system. In mammalian systems, PKG has been implicated in Ca^2+^ mobilization and influx in glia (Willmott, Wong, & Strong, 2000), and in translocation to the nucleus to regulate gene expression in glia (Gudi, Hong, Vaandrager, Lohmann, & Pilz, 1999). In *D. melanogaster*, the perineurial and sub-perineurial glia function as the blood–brain barrier and are important for regulating the transport and exchange of nutrients, metabolites, and hormones between the brain and the hemolymph (Stork *et al.*, 2008). *foraging* may affect behaviors, such as learning and memory, in the fly by regulating the Ca^2+^ environment surrounding neuronal cell bodies via its cortex glial expression. Cortex glia, in the fly, have been shown to have Ca^2+^ oscillations which when disrupted can alter behavior (Melom & Littleton, 2013). Alternatively, *foraging* may alter the metabolic environment of the brain by mediating nutrient and waste transport via its perineurial glia expression. Further experiments are required to test this hypothesis.

Although we found strong evidence that *foraging* is expressed in glia of the adult brain, we found no detectable neuronal expression in the adult brain using the *for^CR00867-TG4.2^* allele (Figure 1(B–D)) and the single-cell transcriptomics data (Figure 2(A–D)). In contrast, *foraging* is expressed in some peripheral neurons and central neurons at earlier stages of development. *foraging* was detected in adult photoreceptor neurons (Davie *et al.*, 2018; Davis *et al.*, 2020; Figure 2(D)), the 3rd instar larval neuromuscular junction (Dason *et al.*, 2019), and in the VNC along the midline of late-stage embryos (Peng *et al.*, 2016). Many studies have demonstrated a role for PKG in mammalian nervous systems (reviewed in Feil, Hofmann, & Kleppisch, 2005; Hofmann, Feil, Kleppisch, & Schlossmann, 2006). For example, cerebellum specific conditional knockout of PKG in the mouse impairs long-term depression affecting motor learning (Feil *et al.*, 2003), and a nociceptor specific knockout in the dorsal root ganglia abolishes long-term potentiation affecting pain sensitivity (Luo *et al.*, 2012). *Prkg1*, *foraging*’s mouse homologue, is readily detected in many subtypes of neurons and glia in the brain of both the developing mouse embryo and adolescent mouse, using single-cell RNA-Seq (La Manno *et al.*, 2020; Zeisel *et al.*, 2018). The readily detectable neuronal expression in the mouse may suggest some differences in regulation between the fly and the mouse. The expression was strongest in the neuroblasts of the developing embryo (La Manno *et al.*, 2020) and the enteric and sensory neurons of the peripheral nervous system in the adolescent mouse (Zeisel *et al.*, 2018). Taken together, it is possible that *foraging*, in *D. melanogaster*, may play a stronger role in the developing nervous system and the developed peripheral nervous system then it does in the adult central brain.

We also found that our previously published anti-FOR antibody (Belay *et al.*, 2007) is not an effective tool for immunohistochemical analyses of FOR in the adult brain. Problems with the unreliability of antibodies have been well discussed in the literature (Egelhofer *et al.*, 2011; Fritschy, 2008; Michel, Wieland, & Tsujimoto, 2009; Rhodes & Trimmer, 2006; Saper & Sawchenko, 2003). We re-evaluated the efficacy of immunohistochemistry of a previously published (Belay *et al.*, 2007) anti-FOR antibody using a complete null allele of *foraging* (*for*^0^; Allen *et al.*, 2017). We found that the anti-FOR antibody labels *bone fide* FOR expression in cells with morphology consistent with the outer optic chiasm glia. However, the anti-FOR antibody had extensive nonspecific immunoreactivity in neurons, including mushroom and ellipsoid bodies. We concluded that the anti-FOR antibody known to be a valuable tool on westerns (Allen *et al.*, 2017; Dason *et al.*, 2019) is not effective for immunohistochemical analyses of FOR in the adult brain. Data from single-cell RNA-Seq supported the findings of glial expression, but no neuronal expression, seen in the *for^CR00867-TG4.2^* allele. We cannot, however, distinguish between a very low expression of *foraging* or no expression of *foraging* outside of glia in the adult brain. This is discussed further, below.

Previous studies suggest that *foraging* functions in neurons to affect adult behaviors, including various forms of adult learning and memory (Kuntz *et al.*, 2012; Mery *et al.*, 2007; Wang *et al.*, 2008), olfactory startle habituation (Eddison *et al.*, 2012), movement in an open field (Burns *et al.*, 2012) and sleep (Donlea *et al.*, 2012). Many of these studies relied on targeting a *UAS-for^cDNA^* pan-neuronally, or to specific subsets of neurons from multiple regions of the brain, including antennal lobe, mushroom bodies, and central complex. These studies, which use a *UAS-for^cDNA^*, clearly show that FOR protein can alter multiple behaviors when expressed in these neuronal populations. What is not clear is whether *foraging* is naturally expressed in the cells in these above studies. Two of the above studies, however, took advantage of *UAS-for^RNAi^* (Donlea *et al.*, 2012; Kuntz *et al.*, 2012). Donlea *et al.* (2012) showed that driving *UAS-for^RNAi^* in the α/β Kenyon cells, with the *30Y* enhancer trap, altered short term memory when compared with controls. Kuntz *et al.* (2012) altered visual orientation memory by driving *UAS-for^RNAi^* in Kenyon cells and ring neurons, with the *lilli^189Y^* enhancer trap. This strengthens the case for *bone fide foraging* expression in these respective cells, although, RNAi can have off-target effects (Ma *et al.*, 2006; Moffat *et al.*, 2007).

Many of these studies relied on the previously published anti-FOR pattern in the adult brain (Belay *et al.*, 2007; Mery *et al.*, 2007) which we show in this study to be non-specific (Figure 3). It is important to note that non-specific binding of the anti-FOR antibody does not necessarily mean that *foraging* is not expressed in neurons such as the Kenyon cells of the mushroom bodies or the ring neurons of the central complex. One of the above studies showed that driving a *UAS-for^cDNA^* in the R3 ellipsoid body ring neurons affects working memory in a visual orientation paradigm (Kuntz *et al.*, 2012). In this case, a number of *Gal4* enhancer trap lines were used, targeting subsets of the ellipsoid body ring neurons, and mushroom body Kenyon cells. One such enhancer trap was *lilli^189Y^* (previously referred to as *for^189Y^*) that was initially thought to be inserted in *foraging* (Osborne *et al.*, 1997) but was subsequently shown to be inserted in *lilliputian* (Sokolowski 2007 FlyBase update FBrf0198702; Wang *et al.*, 2008). Since *lilli^189Y^* is not inserted in *foraging*, we cannot assume that it would necessarily recapitulate *foraging* expression.

Another of the above studies used enhancer traps in *foraging* to infer the expression of *foraging* in the adult brain (Eddison *et al.*, 2012). Eddison *et al.* (2012) used two enhancer trap lines, *for^11.247^* and *for^2614^*, which were inserted in the *foraging* gene at its most 5′ end near the *for^pr1^* TSS. At the time, parts of the expression of *for^11.247^* were validated with the anti-FOR antibody. The enhancer traps in *foraging* and the promoter fusions from *foraging*, used in the present study, are equally likely to reflect actual *foraging* expression and may represent distinct subsets of expression in the adult CNS. However, it is also possible that these 5′ insertions may trap enhancers of the neighbouring gene, *Drgx*, which has been shown to contain regulatory elements that drive expression in the antennal lobe, mushroom bodies, as well as the ellipsoid body (Jenett *et al.*, 2012). The possibility of crosstalk with *Drgx* enhancers may also exist for our *for^pr1^-Gal4* as this cloned region lies between *foraging* and *Drgx*.

There is an apparent discrepancy between the lack of detectable *foraging* expression in adult brain neurons found in the present study and published papers showing that transgenically expressing *foraging*, with a *UAS-for^cDNA^*, in adult brain neurons alters behavioral phenotypes. Some possible reasons for this are as follows: 1) *foraging* is expressed in these tissues, but at levels too low to be detected with the methods used in the present study. 2) *foraging* is only conditionally expressed in these neuronal populations depending on certain environmental conditions. 3) *foraging* is expressed in these neurons, but only during development. 4) These previous studies using *UAS-for^cDNA^* represent ectopic expression, driving FOR protein where it is not naturally expressed. We discuss these possibilities below.

To attempt to address the sensitivity issue of single-cell RNA-Seq, we looked for *foraging* expression in bulk RNA-Seq studies, which are more sensitive, on sorted neuronal cell populations. Three separate studies, in which multiple sub-populations of Kenyon cells were assayed, failed to detect *foraging* in Kenyon cells at levels higher than that of *repo*, or other glial-specific or non-neuronal markers (Crocker, Guan, Murphy, & Murthy, 2016; Davis *et al.*, 2020; Shih, Davis, Henry, & Dubnau, 2019). Davis *et al.* (2020) did however find *foraging* expression in photoreceptor neurons in the eye of the fly, which was also seen in single-cell transcriptomic data (Davie *et al.*, 2018; Figure 2(D)). Mammalian PKG has previously been shown to be involved in neurite plasticity in rod and cone cells (Zhang, Beuve, & Townes-Anderson, 2005).

We currently do not know whether *foraging* is conditionally expressed in adult brain neurons in different environmental contexts, such as different nutritional states or different learning paradigms. *foraging* expression has previously been shown to be up regulated by food deprivation in whole adult heads (Kent *et al.*, 2009). Although, the head is more than just neurons, and so fat cells, trachea, and glia in the head capsule are also candidate cell types for this observed effect. Further experiments specifically assaying adult brain neurons in differing environmental conditions are required to explicitly test any potential condition expression of *foraging*.

We currently do not have any direct measures of *foraging* expression in neurons during different developmental stages. However, two of the above-mentioned studies (Kuntz *et al.*, 2012; Wang *et al.*, 2008) did employ the temperature sensitive GAL4 repressor GAL80^ts^ (McGuire *et al.*, 2003) to restrict expression of the *UAS-for^cDNA^* to post-eclosion adults. In both cases, the authors found that adult restricted expression was sufficient to modulate their respective phenotypes, working memory in a visual orientation paradigm (Kuntz *et al.*, 2012) and visual pattern memory (Wang *et al.*, 2008). In these cases, the developmental possibility is, at least in part, ruled out. Again, further experiments are required to assess the potential role for *foraging* in neurons of developing larvae and pupae.

Finally, if in fact, *foraging* is not naturally expressed in the adult brain neurons where *UAS-for^cDNA^* was expressed in previous studies, then the *UAS-for^cDNA^* manipulations constitute ectopic expression. In this case, FOR protein may still be interacting with *bone fide* PKG targets, which may typically co-express in other cell types. The observed effects may then represent neomorphic-like phenotypes. Alternatively, at the biochemical level there can be crosstalk between the cAMP and cGMP systems, whether by PKG activation by cAMP (Lin, Liu, Chow, & Lue, 2002; Lin, Liu, Tu, Chow, & Lue, 2001; Lincoln & Cornwell, 1991; Ruiz-Velasco, Zhong, Hume, & Keef, 1998; White, Kryman, El-Mowafy, Han, & Carrier, 2000), or PKG phosphorylating a PKA target due to similar phosphorylation target recognition sites (Douglass *et al.*, 2012), or even by PKG phosphorylating shared target proteins (Döppler & Storz, 2013; Huang, Tsai, Chen, Wu, & Chen, 2007). More research is needed into the precise cellular localization and expression of *foraging* to decipher how its expression influences each of the gene’s pleiotropic phenotypes.

### Foraging in the gastric and reproductive systems

There are many potential roles for *foraging* in the gastric and reproductive systems. The visceral muscle of the gut is required to push nutrients along via peristalsis. Mice with *foraging*'s orthologue knocked out in smooth muscle show increased gut passage times relative to controls (Weber *et al.*, 2007). *foraging* has been implicated fecal excretion rate in adults (Urquhart-Cronish & Sokolowski, 2014) and the visceral muscle is a candidate tissue to mediating this function. *foraging* has also been implicated in the rate of glucose absorption in larvae (Kaun *et al.*, 2007). Simple sugars are absorbed by the enterocytes of the midgut (reviewed in Miguel-Aliaga *et al.*, 2018) and so are strong candidate cells for *foraging*’s glucose absorption phenotype. *foraging* has also previously been shown to affect adult Malpighian tubule secretion rate (MacPherson, Broderick, *et al.*, 2004; MacPherson, Lohmann, & Davies, 2004) and the expression in the principal cells is consistent with this phenotype.

As for the reproductive systems, differences in the number of eggs laid by flies reared in differing nutritional conditions during development (Burns *et al.*, 2012), as well as oviposition site selection (McConnell & Fitzpatrick, 2017) has previously been associated with *foraging*. The expression seen in the spermatheca and visceral muscle of the reproductive system represent candidate cells for mediating this difference. The spermathecae are essential for long term storage of sperm (Gilbert, 1981), and perturbations in these cells can cause a decrease in egg-laying over time (Schnakenberg, Matias, & Siegal, 2011). *foraging* has also been shown to play a role in developmental death and cell clearance in the epithelial follicle cells of the *D. melanogaster* ovary under starvation conditions (Lebo *et al.*, 2021). The maternal loading of *foraging* in the developing eggs and the early embryo is also well known (Graveley *et al.*, 2011; Jambor *et al.*, 2015; Koenecke *et al.*, 2016; Tomancak *et al.*, 2002), suggesting a role for *foraging* in very early development.

### Foraging’s complexity and the mapping of CREs

Three genes, *foraging, Pkg21D,* and *CG4839* code for a cGMP-dependent protein kinase (PKG) in *D. melanogaster* (Thurmond *et al.*, 2019). *foraging* is by far the most complex locus. *Pkg21D* is less than 5 kb with 1 TSS, 1 transcript and 1 protein isoform, while *CG4839* is less than 12 kb with 1 TSS, 2 transcripts, and 1 protein isoform (Thurmond *et al.*, 2019). In contrast, *foraging* is a 35 kb locus with 4 TSSs, more than 20 transcripts, and potentially more than 9 protein isoforms (Allen *et al.*, 2017). The spatial and temporal expression of the *Pkg21D* and *CG4839* is much more restricted than that of *foraging*, mirroring their relative complexities. *Pkg21D* is primarily expressed in hindgut and Malpighian tubules, and *CG4839* is primarily expressed in the midgut, salivary gland, and reproductive tissues (Leader *et al.*, 2018). Neither appear to be detected in the adult brain (Davie *et al.*, 2018; Leader *et al.*, 2018). *foraging* orthologues play multiple roles in behavioral and physiological phenotypes in multiple taxa (reviewed in Anreiter & Sokolowski, 2019; reviewed in Reaume & Sokolowski, 2009).

A clue to *foraging*’s complexity may reside in the structure of *foraging*’s promoters and the mapped CREs. Core promoters are described by two different shapes; “narrow” (a.k.a. “peaked”, “focused”, “sharp”) promoters with transcription always initiating at a within a narrow range of a few bases, and “broad” (a.k.a. “dispersed”) promoters with transcription initiating over a larger range of bases (Haberle & Stark, 2018). Narrow promoters tend to have more spatially and temporally restricted expression patterns, whereas broad promoters tend to be more ubiquitous (Bhardwaj, Semplicio, Erdogdu, Manke, & Akhtar, 2019; Hoskins *et al.*, 2011; Schor *et al.*, 2017). Three of *foraging*’s promoters, *for^pr1^*, *for^pr2^*, and *for^pr4^*, are narrow promoters and one, *for^pr3^*, is broad (Allen *et al.*, 2017; Hoskins *et al.*, 2011). Based on the *for^pr^-Gal4* expression patterns reported here, the CRE driving the secondary cells of the male accessory glands mapped very close to the TSS of *for^pr1^*, *for^pr2^*, and *for^pr4^*. This closely linked accessory gland CRE and the fact that *for^pr1^*, *for^pr2^*, and *for^pr4^* are all narrow promoters may suggest a common evolutionary origin of these TSSs.

Promoter analyses has been successfully used previously to identify CREs and deduce isoform-specific expression and function in many genes (Arredondo *et al.*, 2001; Billeter & Goodwin, 2004; Brenner, Thomas, Becker, & Atkinson, 1996; Lehman *et al.*, 1999; Okada *et al.*, 2001; Park *et al.*, 2000). Consequently, we used a promoter analysis strategy to map multiple CREs along the *foraging* locus responsible for a subset of the expression seen in the *T2A-Gal4* gene-trap allele. The expression seen in the *for^pr^-Gal4*s may be only a subset of that seen in the *for^CR00867-TG4.2^* allele because the regions cloned into the *for^pr^-Gal4*s encompass only 15 kb of the 35 kb locus, suggesting that some CREs were missed and/or because each *for^pr^-Gal4* was inserted outside the context of the *foraging* locus at a common site on the third chromosomes (see “Methods”).

### Conserved expression and regulation across development

We can draw parallels between the regulation, expression, and function of genes across development from larva to adult despite the differences in life-history traits of these developmental stages. Many genes show consistent expression across development (Graveley *et al.*, 2011). Additionally, promoter structure can be highly conserved between developmental stages (Hoskins *et al.*, 2011). Like *foraging,* the CREs of *slowpoke*, *pigment-dispersing factor*, and *paramyosin* drive consistent expression across development (Arredondo *et al.*, 2001; Brenner & Atkinson, 1996; Park *et al.*, 2000). The expression levels and spatial distribution of *foraging* are similar between tissues of the third instar larva and adult fly, as deduced from microarray and RNA-Seq on dissected tissues (Chintapalli *et al.*, 2007; Leader *et al.*, 2018). Here we show that many but not all the CREs within the *foraging* locus also drive consistent expression between these two stages. The parallels in the expression of *foraging* at these two developmental stages mirror many of the parallels seen in the larval and adult phenotypic traits.

## Conclusion

Our characterized expression patterns of *foraging* provide novel candidate tissues and cell types to explore *foraging*’s pleiotropic influences on physiology and behavior. We provide exciting new data on *foraging* expression in multiple subsets of glia in the adult brain, paving the way towards functional studies that address the importance of glial subtypes in each of *foraging*’s suite of pleiotropic behavioral phenotypes.

## Supplementary Material

Supplemental Material

Supplemental Material
