# Supplementary figures and images for: Expression of the foraging gene in adult Drosophila melanogaster
Source: J Neurogenet. 2021 Aug 12;35(3):192–212. doi: 10.1080/01677063.2021.1941946 (PMC8846931; doi:10.1080/01677063.2021.1941946)

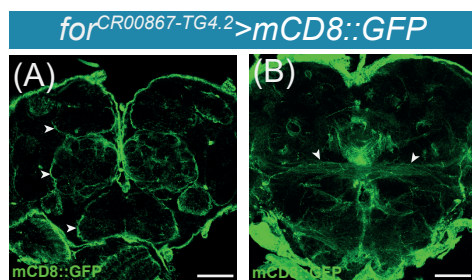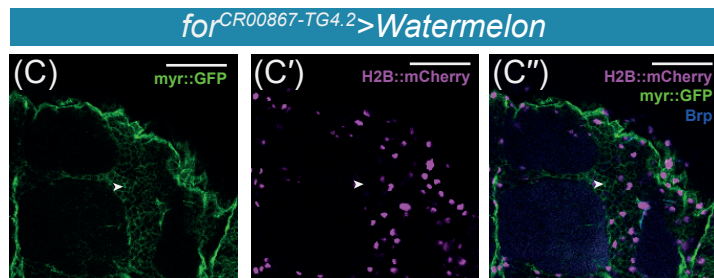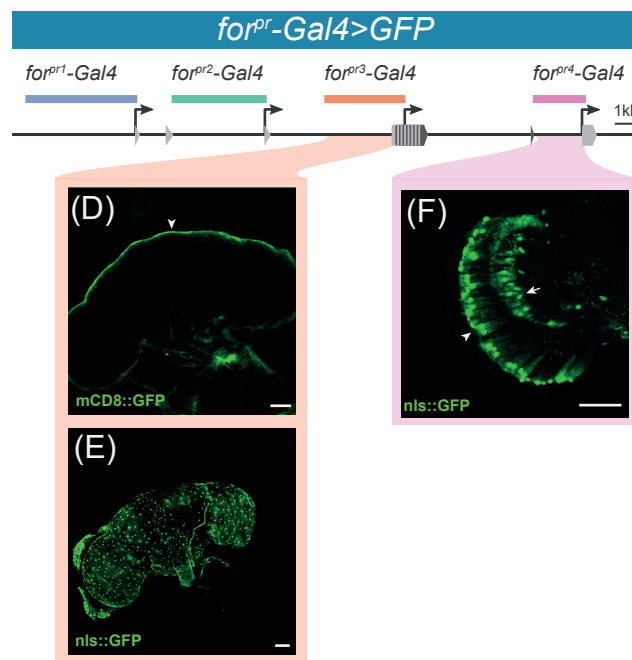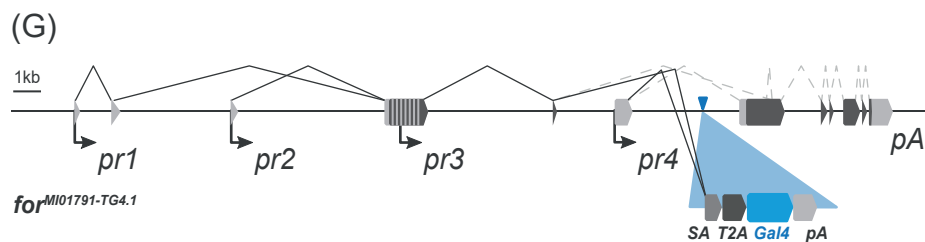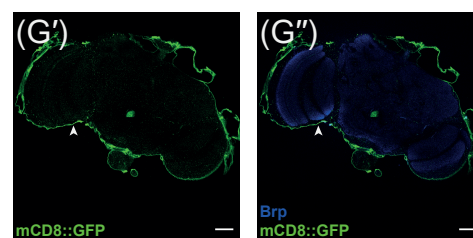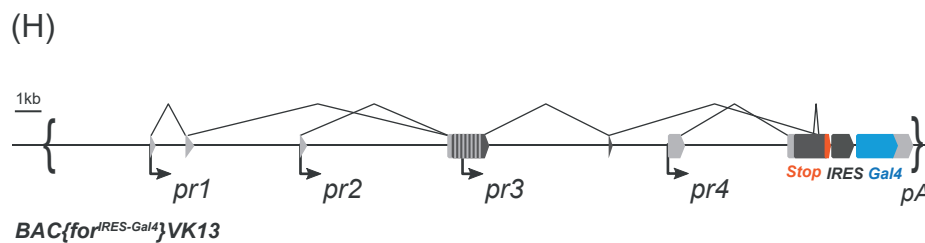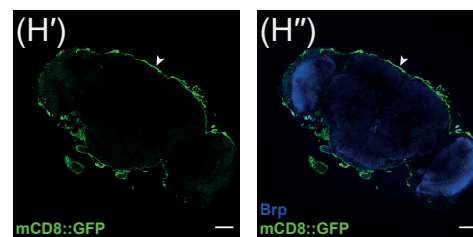

Figure S1

Supplement: Supplemental Material [file INEG_A_1941946_SM8916.pdf]

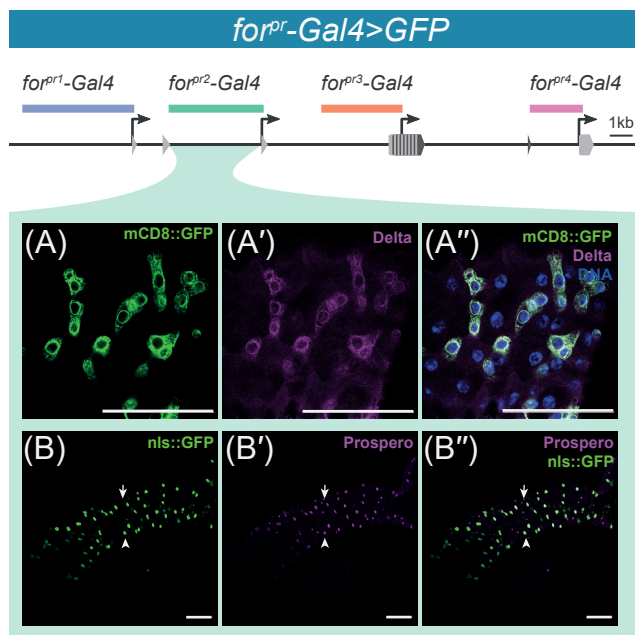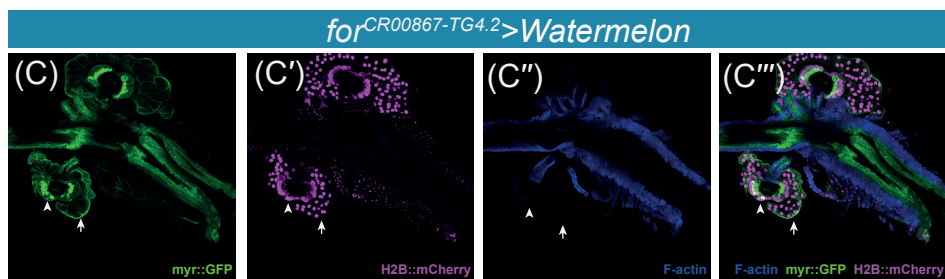

Figure S2

Supplement: Supplemental Material [file INEG_A_1941946_SM7149.pdf]
